# Direct replication of task‐dependent neural activation patterns during sadness introspection in two independent adolescent samples

**DOI:** 10.1002/hbm.24836

**Published:** 2019-10-22

**Authors:** Veronika Vilgis, Shawn A. Rhoads, David G. Weissman, Kristina L. Gelardi, Erika E. Forbes, Alison E. Hipwell, Kate Keenan, Paul D. Hastings, Amanda E. Guyer

**Affiliations:** ^1^ Center for Mind and Brain University of California Davis California; ^2^ Department of Psychology Georgetown University Washington District of Columbia; ^3^ Department of Psychology University of California Davis California; ^4^ Department of Human Ecology University of California Davis California; ^5^ Department of Psychiatry University of Pittsburgh Pittsburgh Pennsylvania; ^6^ Department of Psychiatry and Behavioral Neuroscience University of Chicago Chicago Illinois

**Keywords:** adolescence, attention, emotional faces, fMRI, introspection, replication

## Abstract

Functional neuroimaging results need to replicate to inform sound models of human social cognition and its neural correlates. Introspection, the capacity to reflect on one's thoughts and feelings, is one process required for normative social cognition and emotional functioning. Engaging in introspection draws on a network of brain regions including medial prefrontal cortex (mPFC), posterior cingulate cortex (PCC), middle temporal gyri (MTG), and temporoparietal junction (TPJ). Maturation of these regions during adolescence mirrors the behavioral advances seen in adolescent social cognition, but the neural correlates of introspection in adolescence need to replicate to confirm their generalizability and role as a possible mechanism. The current study investigated whether reflecting upon one's own feelings of sadness would activate and replicate similar brain regions in two independent samples of adolescents. Participants included 156 adolescents (50% female) from the California Families Project and 119 adolescent girls from the Pittsburgh Girls Study of Emotion. All participants completed the Emotion Regulation Questionnaire (ERQ) and underwent a functional magnetic resonance imaging scan while completing the same facial emotion‐processing task at age 16–17 years. Both samples showed similar whole‐brain activation patterns when engaged in sadness introspection and when judging a nonemotional facial feature. Whole‐brain activation was unrelated to ERQ scores in both samples. Neural responsivity to task manipulations replicated in regions recruited for socio‐emotional (mPFC, PCC, MTG, TPJ) and attention (dorsolateral PFC, precentral gyri, superior occipital gyrus, superior parietal lobule) processing. These findings demonstrate robust replication of neural engagement during sadness introspection in two independent adolescent samples.

## 1 INTRODUCTION

Replication is critical to developing solid conceptual models of human behavior and its neural correlates. However, surprisingly few examples of direct replication of functional neuroimaging results have been published (Poldrack et al., 2017). The scarcity of such replication efforts is also evident in social cognitive neuroscience research using child and adolescent samples. Although few direct replications exist, examinations of resting state networks have shown generally stable patterns of blood oxygen‐level‐dependent (BOLD) signal activation and meta‐analyses of task‐based activation patterns have identified reliable neural correlates for various psychological processes measured using cognitive tasks (Gilmore, Diaz, Wyble, & Yarkoni, 2017). Nonetheless, as Gilmore et al. (2017) indicate, “it is one thing to establish that neuroimaging methods can consistently reveal broad mappings between cognitive processes and distributed brain networks, and quite another to establish that the specific pattern of findings generated by any single study can be reproduced with a high degree of fidelity in another study” (p. 9). The current study sought to address this need by identifying whole‐brain neural activation during a social cognition task involving emotion introspection in one sample of adolescents and testing whether the pattern replicated in an independent second sample of adolescents.

Facial expressions play a key role in conveying information about our own and others' emotional states. The ability to recognize and interpret others' emotional states from facial expressions and subsequently appraise one's own emotions and behaviors are thus crucial skills for successful social interaction, which is a primary developmental task of adolescence. Accordingly, adolescence provides a prolonged period for learning complex social and emotion regulation skills that are necessary for mature interpersonal communication (Crone & Dahl, 2012; Nelson, Jarcho, & Guyer, 2016). This period also overlaps with the protracted course of development of the prefrontal cortex (PFC) (Giedd et al., 1999; Lenroot & Giedd, 2006), a critical brain region that supports central cognitive control processes including the regulation of emotion, decision making and mentalizing, and thus undergirds many complex social and emotional skills.

Becoming skilled in interpersonal communication is also a dynamic process. Another person's affective expressions may prompt our own affective states to change, triggering a need for self‐regulation and influencing subsequent behavior. Directing attention to one's own feelings and thoughts, also known as introspection, is hypothesized to emerge through interactions with others (Rimé, 2009). This observation is corroborated by evidence of a close link between brain regions involved in reflecting upon one's own thoughts and emotions and those engaged by social cognition tasks (Schilbach et al., 2012). Introspection emerges in late childhood and early adolescence when children start to attribute emotions to an internal state rather than external circumstances (Harris, Olthof, & Terwogt, 1981). During adolescence, increased awareness of one's internal emotional states becomes more integrated with knowledge of appropriate displays of emotion in social situations but is also thought to contribute to increased emotional instability and higher incidences of affective and anxiety disorders during this period (Guyer, Silk, & Nelson, 2016). Accordingly, insights into neural processes underlying introspection may increase our understanding of how to foster emotional and interpersonal competence in adolescents.

Neuroimaging research has identified some of the neural correlates of emotion recognition and introspection. For example, studies of adults show the amygdala, fusiform gyrus, orbitofrontal cortex (OFC), superior temporal gyrus, and somatosensory‐related cortices are activated while processing emotional facial expressions (Adolphs, 2002). Face processing at the most basic level is a prototypical perceptual function that occurs in the occipital face area, fusiform face area, and several other regions across the superior temporal sulcus and inferior temporal lobe (Nelson et al., 2016; Weiner & Grill‐Spector, 2015). Beyond simple perceptual processing, social cognition tasks that require the correct interpretation of facial expressions also activate the temporoparietal junction (TPJ) and insula cortices, extended face processing regions implicated in person knowledge and emotion (Blakemore, 2008; Gobbini & Haxby, 2007; Scherf, Behrmann, & Dahl, 2012).

Furthermore, implicit and explicit processing of emotional stimuli, such as faces, relies on overlapping yet distinct brain systems. For example, implicit processing of emotional stimuli (e.g., identifying a nonemotional feature of a face, such as gender) has been associated with activating a network comprising occipital lobe regions including lingual and fusiform gyri, postcentral gyri, and insula cortices (Fusar‐Poli et al., 2009). In contrast, explicit processing (e.g., identifying the emotion on a face) commonly activates the amygdala and parts of the PFC. Processes involving introspection, self‐referential thought, and social cognition are associated with neural activity in cortical midline structures such as the medial PFC (mPFC) and posterior cingulate cortex (PCC) as well as the TPJ (Denny, Kober, Wager, & Ochsner, 2012; Feng, Yan, Huang, Han, & Ma, 2018; Hu et al., 2016; Lieberman, Straccia, Meyer, Du, & Tan, 2019; Northoff et al., 2006). For example, making judgments about the self has been found to activate ventral portions of the medial and (left) lateral PFC and the left insula whereas making judgments about others has been found to engage dorsal parts of the mPFC, TPJ, and cuneus (Denny et al., 2012). Within samples of adolescents and adults, the same regions are elicited during introspective, self‐referential processing, although direct comparisons between these age groups show overall greater neural activation in both self and other networks in adolescents during self‐appraisal (Pfeifer et al., 2009; Pfeifer, Lieberman, & Dapretto, 2007). The mPFC is found to be activated when adolescents perform tasks that explicitly engage awareness of their own emotions and that require processing complex social interactions (Blakemore, 2008; Kilford, Garrett, & Blakemore, 2016; Sebastian, Burnett, & Blakemore, 2008) supporting observations in adults that indicate self‐referential processing in the emotional domain especially involves anterior cortical midline structures (Northoff et al., 2006).

Given its anatomical connections to parts of the temporal lobe and subcortical structures that control autonomic responses (Fusar‐Poli et al., 2009), the mPFC has direct involvement in monitoring ongoing emotional arousal (McKlveen, Myers, & Herman, 2015). Together, the mPFC with the PCC/precuneus and bilateral inferior parietal lobule (angular gyrus) form a brain network implicated in social cognition and affective processing that converges largely with the default‐mode network (DMN). The DMN tends to be more active in the absence of task demands than during tasks requiring attentional focus (Raichle et al., 2001), but is also involved during mentalizing and self‐directed cognitive processes, such as autobiographical memory retrieval (Raichle et al., 2001), emotion processing (e.g., Sreenivas, Boehm, & Linden, 2012; Wiebking et al., 2011), and depression (e.g., Sheline et al., 2009; Shi et al., 2015).

The present study leveraged a unique opportunity to conduct a direct replication of neural responses during a facial emotion‐processing task in two independent samples of adolescents from diverse ethnic backgrounds. The replication of specific activation patterns in an independent adolescent sample would provide valuable information on the reliability of functional magnetic resonance imaging (fMRI) results in general, and of explicit (introspection) and implicit (facial feature) emotion processing activation patterns, more specifically. We analyzed fMRI data collected from two independent samples of adolescents recruited into neurobiological substudies on social–emotional development and elevated risk for psychopathology: one of Mexican‐origin youth studied from ages 10 to 21 years and one of racially diverse girls assessed from ages 9 to 20 years. Aiding in our replication effort, at age 16–17 years, both samples underwent a functional brain imaging scan while completing a facial emotion‐processing task designed to have participants reflect upon their own state of sadness and judge a face's nose width when viewing different facial expressions.

Using a region of interest (ROI) approach, we have shown that dorsomedial (dmPFC) activity during sadness introspection when viewing sad faces was related to depression severity 1 year later and to self‐reported emotion regulation (Vilgis et al., 2018). We have also found that stronger activation of social–emotional processing regions (i.e., the PCC, left TPJ, and left amygdala) during sadness introspection, regardless of facial expression, moderated the relation between community crime exposure and disruptive behavior problems (Weissman et al., 2018). In accordance with this previous research, our first hypothesis of the present study was that engaging in sadness introspection (explicit emotion processing) would induce specific patterns of neural activity in social–emotional processing and DMN regions, including mPFC, precuneus, and temporal regions, similar to what other studies have shown. We also expected that rating a nonemotional facial feature (implicit emotion processing) would elicit activation in occipital regions, including lingual and fusiform gyri, postcentral gyri and insula cortices. Our second hypothesis of the present study was that these neural activation patterns would be replicated in another independent sample when comparing the activation of significant clusters between the samples. In addition, we explored whether self‐reported emotion regulation strategies were associated with task activation in the two samples.

Due to the scarcity of direct replication studies in the task‐based functional neuroimaging literature, there are few guidelines or best practices for testing whether activation replicates across samples. Therefore, we used several different approaches to assess replicability, with the goal of showing convergence across the approaches. First, we independently modeled activation in each sample, probed for overlapping clusters, and visualized the contrast estimates per cluster for each sample. As the same research group typically does not perform replication studies, the logical first step was to model the two samples separately. Second, we included them in the same model to perform a direct statistical comparison between the two samples. Third, because traditional neuroimaging studies are typically not designed to estimate effect sizes (Reddan, Lindquist, & Wager, 2017), we extracted percent signal change in a priori defined regions of interest to focus on a more direct measure of effect magnitude. Although each approach has its limitations for assessing reproducibility, we aimed to inform future fMRI replication efforts by providing new evidence based on multiple statistical approaches.

## 2 METHOD

### 2.1 Participants

The present study included two samples, one recruited in one midsized and one small city in Northern California and the other in a large metropolitan city in Western Pennsylvania. Table 1 shows basic demographic characteristics for both samples. Although we have previously published results from analyses using this same fMRI task completed within each sample independently, we have not conducted the same analyses reported in this paper in any of our previous publications for either sample (Vilgis et al., 2018; Weissman, Gelardi, et al., 2018; Weissman, Guyer, Ferrer, Robins, & Hastings, 2018).

**Table 1:** Demographic information for the CFP and the PGS‐E samples

	CFP (*n* = 156)	PGS‐E (*n* = 119)
Age at scan, mean (*SD*)	16.1 (0.4)	16.9 (0.6)
Female (male), *n*	77 (79)	119 (0)
Caucasian, *n* (%)	—	32 (26.9)
African American, *n* (%)	—	79 (66.4)
Mexican, *n* (%)	80 (51.3)	—
Mexican American, *n* (%)	75 (48.1)	—
Other ethnicity, *n* (%)	1 (0.6)	8 (6.7)
Receipt of public assistance, *n* (%)^a^	51 (32.7)	66 (55.5)
Single parent household, *n* (%)^a^	36 (23.1)	56 (47.1)
Maternal education >12 years, *n* (%)	61 (39.1)	75 (63.0)
Median income ($US)^a^	$25,000–39,999	N/A
ERQ reappraisal score, mean (*SD*)	29.72 (6.35)	28.50 (7.51)
ERQ suppression score, mean (*SD*)	15.59 (4.76)	13.68 (4.66)

Abbreviations: CFP, California Families Project; PGS‐E, Pittsburgh Girls Study of Emotion.

a Measure was collected within 18 months of the scan; *SD*, standard deviation; N/A, measure was not collected in this study; ERQ, Emotion Regulation Questionnaire.

#### 2.1.1 Sample one

The first sample was drawn from the California Families Project (CFP) (Atherton, Ferrer, & Robins, 2018; Cruz, King, Mechammil, Bamaca‐Colbert, & Robins, 2018; Martin, Bacher, Conger, & Robins, 2018), which included 674 participants of Mexican‐origin. Initially, participants were recruited when they were in fifth grade, drawn at random in two cohorts from school rosters during the 2006–2007 and 2007–2008 school years. About 6 years later, 229 participants (119 males and 110 females) were recruited into a substudy designed to examine neurobiological mechanisms in the etiology of depression (Schriber et al., 2017; Schriber et al., 2018; Weissman, Gelardi, et al., 2018; Weissman, Guyer, Ferrer, Robins, & Hastings, 2019). Youth with elevated depressive symptoms were oversampled from the CFP parent sample, using counts of adolescents' self‐reported symptoms in ninth grade (Age 14) on the Diagnostic Interview Schedule for Children‐IV (Shaffer, Fisher, Lucas, Dulcan, & Schwab‐Stone, 2000) and indicators of elevated severity from the Anhedonic Depression and General Distress subscales of the Mood and Anxiety Symptom Questionnaire (Watson & Clark, 1991). At Age 16, 192 participants completed the facial emotion‐processing task during an MRI scan (eight refused to be scanned and 36 did not complete the face task due to time constraints and/or scanner malfunction) (Weissman, Gelardi, et al., 2018). Of those who completed the scan, exclusion from analyses due to excessive head motion (*n* = 34) or poor understanding of the behavioral task (missed responses to >20% trials; *n* = 2) resulted in a final sample of 156 CFP participants in the current investigation. Excluded participants did not differ significantly from included participants with regard to household composition, receipt of public assistance, maternal education, and gender. All participants and their parents provided written assent/consent to take part in this study and received monetary compensation for participation. All study procedures were approved by the study site's Institutional Review Board.

#### 2.1.2 Sample two

The second sample came from the Pittsburgh Girls Study of Emotion (PGS‐E), part of the larger ongoing longitudinal Pittsburgh Girls Study (PGS; Keenan et al., 2010) that has followed 2,450 girls since ages 5–8 years. A subsample of girls from the youngest PGS cohort (*n* = 232) and their mothers were recruited into the PGS‐E when they were 9 years of age. As in the CFP, PGS‐E participants were oversampled for high depression scores: half the girls had scores in the upper quartile on self‐ and/or parent report of depression symptoms. At Age 16, 147 of the girls completed the facial emotion‐processing task during an MRI scan (38 refused to participate or could not be reached, 22 refused to be scanned or could not be scheduled, 25 were ineligible for scanning at the time of the study due to pregnancy, braces, or other scanning exclusions) (Casement et al., 2014; Romens et al., 2015; Vilgis et al., 2018). Of those who completed the scan, exclusion from analyses due to excessive head motion (*n* = 19), poor scan quality (*n* = 5), neural abnormalities (*n* = 2), or poor understanding of the behavioral task (*n* = 2) resulted in 119 participants included in the current investigation. Maternal education >12 years was more common in those participants included versus excluded in the final sample (59.5 vs. 40.5%); maternal education ≤12 years was more common in excluded than included (44.6% vs. 55.4%) participants. The difference in proportion was significant χ^2^ (1, *N* = 218) = 4.78, *p* = .029. There were no significant differences in race distribution and receipt of public assistance between included and excluded participants. All participants and their mothers provided written assent/consent to take part in this study and received monetary compensation for participation. All study procedures were approved by the study site's Human Research Protection Office.

### 2.2 Measures

#### 2.2.1 Emotion regulation

Participants in both studies completed the Emotion Regulation Questionnaire (ERQ; Gross & John, 2003) on the same day they completed the MRI scan. The ERQ measures the habitual use of two different emotion regulation strategies: cognitive reappraisal and expressive suppression. The ERQ consists of 10 items rated on a scale from 1 (strongly disagree) to 7 (strongly agree). An example item from the cognitive reappraisal scale is: “*When I want to feel less negative emotion, I change the way I'm thinking about the situation*.” and from the suppression scale: “*When I am feeling negative emotions, I make sure not to express them*.” Evidence has shown good predictive validity for emotion regulation, such as the ability to downregulate anger (Mauss, Cook, Cheng, & Gross, 2007). Cronbach's alpha for the 6‐item reappraisal scale was .80 and .69 for the 4‐item suppression scale for the CFP sample and .84 and .64 for the PGS‐E sample, respectively.

#### 2.2.2 Facial emotion processing fMRI task

**Figure 1:**
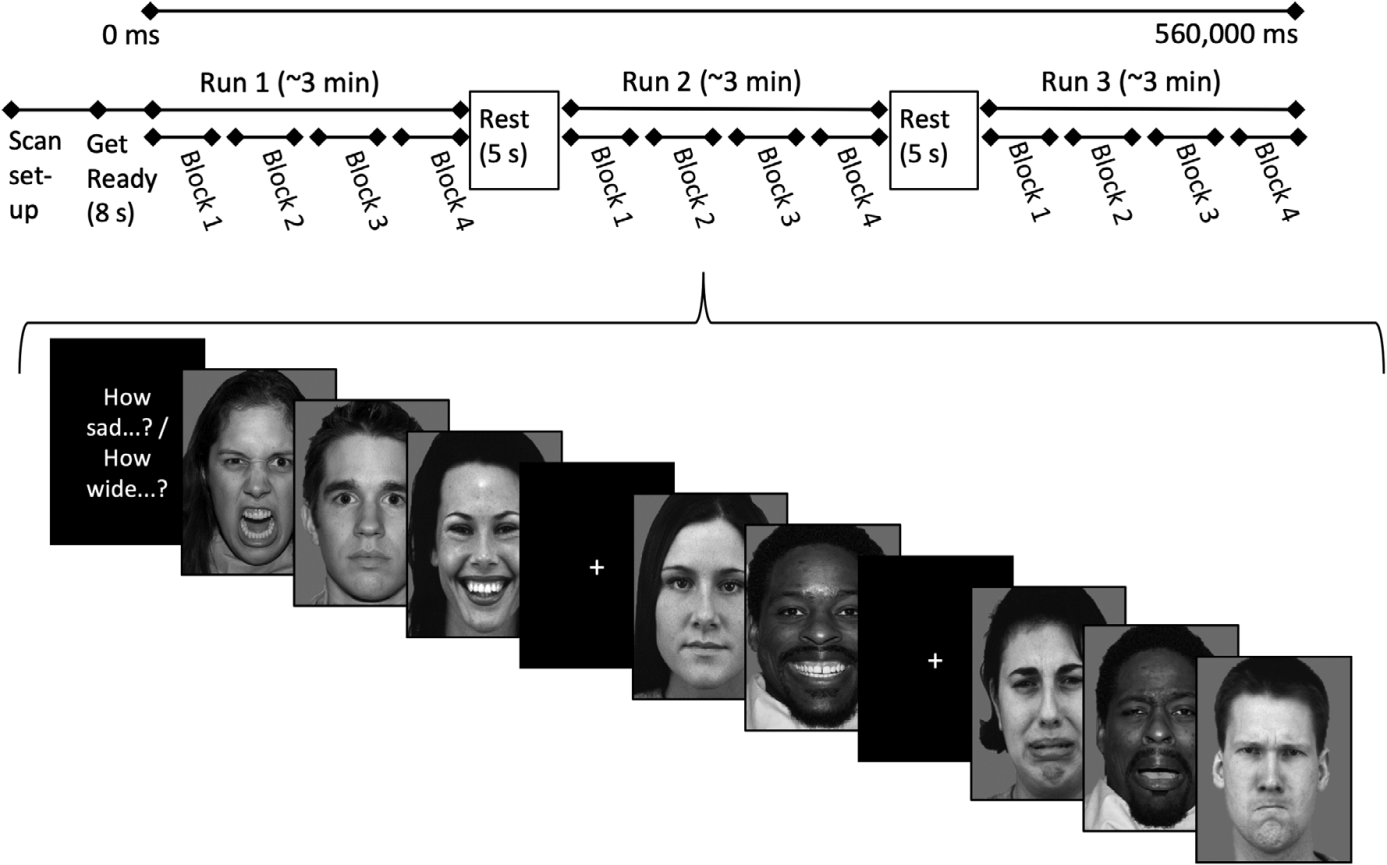
Schematic representation of the facial emotion processing functional magnetic resonance imaging (fMRI) task design. The 9 min 20 s task had three runs, which each contained four blocks. Each block began with an instruction screen (displayed for 4 s) asking participants to rate “How sad does this face make you feel?” or “How wide is the nose?” (1 = Not at all to 5 = Very much so). After the instructions, 10 randomly ordered trials (each displayed for 3 s) depicted eight emotional facial expressions (2 happy, 2 sad, 2 angry, 2 neutral) and two fixation crosses. Intertrial intervals were 750–1,250 ms (not depicted), averaging 1 ms within a block

A facial emotion‐processing task (Guyer et al., 2008; Guyer, Choate, Grimm, Pine, & Keenan, 2011) was used to assess BOLD response to facial expressions of emotion. In this rapid, event‐related fMRI task, participants viewed 12 sad, 12 angry, 12 happy, and 12 neutral faces portrayed by 48 unique actors selected from several databases of emotional faces (Schmidt, Davis, & Tone, 2012; Ebner, Riediger, & Lindenberger, 2010; Lundqvist, Flykt, & Öhman, 1998; Minear & Park, 2004; Nelson, 2004; Tottenham et al., 2009). Each actor's face was presented only once to each participant, displaying one of the four emotions at random, but across participants all actors were displayed with all four expressions. While viewing each picture, participants were asked to direct their attention to either judging “How sad does this person make you feel?” (attention condition: sadness introspection) or “How wide is the nose?” (attention condition: nonemotional judgment of physical feature). In the current study, we focused specifically on BOLD response during sadness introspection versus nonemotional judgment while participants viewed each of the facial expressions. Behavioral responses were recorded via a button box with five buttons, one for each finger, and ranged from 1 = Not at all to 5 = Very much so. Each of the two task conditions began with an instruction screen presented for 4,000 ms. Following the instruction screen, 10 randomly ordered stimulus event trials (eight faces, two fixation crosses) were each presented for 3,000 ms. The two fixation crosses were included to avoid potential collinearity between stimuli. Following each event, an intertrial interval displayed a blank screen that varied from 750 to 1,250 ms (averaging 1,000 ms within a 10‐trial block) to reduce the degree to which participants could predict onset of each face‐viewing event. Presentation order of attention conditions and of facial expressions was randomized across participants. The total duration of the task was 9 min and 20 s consisting of three runs of four 10‐trial blocks. Figure 1 shows a schematic representation of the task design.

### 2.3 MRI acquisition

For both samples, MRI data were acquired on 3.0 T Siemens TIM Trio MRI scanners (Erlangen, Germany), located on the medical campus of each study site. The functional scan for the CFP sample consisted of 275 contiguous T2‐weighted echo planar imaging whole‐brain functional volumes with the following parameters: repetition time (TR) = 2,000 ms; echo time (TE) = 27 ms; flip angle = 80°, 35 slices, matrix = 64 × 64; field of view (FOV) = 224 mm; acquisition voxel size = 3.5 × 3.5 × 3.5 mm^3^. A T1‐weighted high‐resolution anatomical image was acquired for coregistration and normalization of functional images with the following parameters: TR = 2,500 ms; TE = 4.33 ms; flip angle = 7°; 160 slices; FOV = 243 mm; acquisition voxel size = 0.9 × 0.9 × 0.9 mm^3^. The functional scan for the PGS‐E sample differed minimally from that of the CFP sample; images were T2‐weighted and comprised 280 contiguous echo planar imaging whole‐brain functional volumes (TR = 2,000 ms; TE = 28 ms; flip angle = 90°, 39 slices, matrix = 64 × 64; FOV = 205 mm; acquisition voxel size = 3.2 × 3.2 × 3.1 mm^3^). A T1‐weighted high‐resolution anatomical image was acquired with the following parameters: TR = 2,300 ms; TE = 2.98 ms; flip angle = 9°; 160 slices; FOV = 256 mm; acquisition voxel size = 1.0 × 1.0 × 1.2 mm^3^.

### 2.4 Image preprocessing and analysis

Preprocessing and analysis of imaging data were conducted using Statistical Parametric Mapping software (SPM8; http://www.fil.ion.ucl.ac.uk/spm). Functional images were slice time corrected to the acquisition time of the middle slice of each volume, spatially realigned to the first volume in the time series to correct for head motion, and spatially normalized to Montreal Neurological Institute (MNI) stereotaxic space using a 12‐parameter affine model and smoothed using a Gaussian filter set at 6 mm full‐width half maximum. Voxel‐wise signal was ratio‐normalized to the whole brain global mean. Artifact Detection Toolbox (ART; http://www.nitrc.org/projects/artifact_detect/) was used to detect functional movements greater than three *SD* from an individual's mean, more than .5 mm translational and more than .01° of rotation scan‐to‐scan movement. For data to be included in the final analysis, no more than 20% of the volumes could show head movement. Temporal censoring based on ART output was used to remove motion artifacts (Siegel et al., 2014).

For first‐level processing, stimulus onset times for each attention condition and facial emotion were implicitly modeled against rest. Motion estimates derived during preprocessing were included in the individual subject general linear model as covariates of no interest. Whole‐brain analyses were conducted using a 2 (attention condition: introspection, nonemotional judgment) × 4 (facial emotion: sad, angry, happy, neutral) repeated‐measures analysis of variance (ANOVA) with a flexible factorial design independently for each sample to examine main effects of attention condition, facial emotion, and the attention × facial emotion interaction. A family wise error (FWE) corrected threshold of *p* < .05 with >10 voxels per cluster was applied to all analyses. MNI coordinates of activations were provided by SPM, with anatomical labeling obtained from Anatomy Toolbox via bspmview (Eickhoff et al., 2005).

### 2.5 Replication analyses

We used three different approaches to directly compare results between the two samples. For replication Approach 1, we conducted a flexible factorial analysis at the whole‐brain level within each sample separately. We then conducted a conjunction analysis whereby we used each sample's whole brain statistical map to create a conjunction map to assess whether any voxels were common to both samples (see Figure S1 and Table S1 in Supplementary Material). For visualization purposes, we plotted these results to depict the overall variance between the two groups. For replication Approach 2, we conducted a whole‐brain between group analysis (i.e., both samples were included in the same model) using a two‐sample *t* test to compare the samples on the two attention conditions (introspection vs. nonemotional judgment). For replication Approach 3, we tested for between‐group differences in signal change within seven a priori defined functional ROIs based on previously reported BOLD activation related to self‐referential processing (introspection was not available as a searchable term). To define the ROIs, we used Neurosynth‐automated meta‐analysis (http://www.neurosynth.org; Yarkoni, Poldrack, Nichols, Van Essen, & Wager, 2011), which employs a lexical automated meta‐analytic approach to produce maps consistent with those in published meta‐analyses for several terms and concepts (Yarkoni et al., 2011). ROIs were restricted to only those voxels in which the reverse inference prediction exceeded a *t*‐score of 4 or higher and contained >100 voxels. This resulted in seven ROIs: mPFC (963 voxels, center of mass MNI *x*, *y*, *z* coordinates [CM] = −6, 52, 5), PCC (406 voxels, CM = −6, −56, 26), left medial temporal gyrus (MTG) (264 voxels, CM = −58, −12, −20), left TPJ (172 voxels, CM = −48, −66, 32), dmPFC (142 voxels, CM = −10, 42, 40), left inferior temporal gyrus (ITG; 133 voxels, CM = −44, −2, −38), and right TPJ (126 voxels, CM = 44, −62, 24). Marsbar (Brett, Anton, Valabregue, & Poline, 2002) was used to extract percent signal change within each ROI during the introspection condition for each facial expression. Average percent signal change was directly compared between the two samples using two‐sample *t*‐tests.

### 2.6 Statistical analyses of behavioral performance

Task performance (i.e., reaction times, ratings) was analyzed in SPSS v24. For reaction times, we conducted a 2 (attention condition: introspection, nonemotional judgment) × 4 (facial emotion: sad, angry, happy, neutral) repeated‐measures ANOVA. Ratings were analyzed separately for each condition. Adjusted degrees of freedom based on Greenhouse–Geisser correction for violation of sphericity are reported where applicable. These results are presented in the Supplementary Material (see Table S2 and Figure S2).

## 3 RESULTS

Table 1 shows mean (*SD*) cognitive reappraisal and expressive suppression scores from the ERQ for the CFP sample and the PGS‐E sample. The two samples differed significantly in expressive suppression, *t*(270) = 2.99, *p* = .003, but not reappraisal, *t*(270) = 1.45, *p* = .16; significant differences in expressive suppression remained when comparing only the CFP girls with the PGS‐E girls. Thus, the replication analyses presented below were also conducted with gender and expressive suppression as covariates when applicable.

### 3.1 Replication Approach 1: Within‐sample whole‐brain functional activations and conjunction analysis

#### 3.1.1 CFP sample

**Figure 2:**
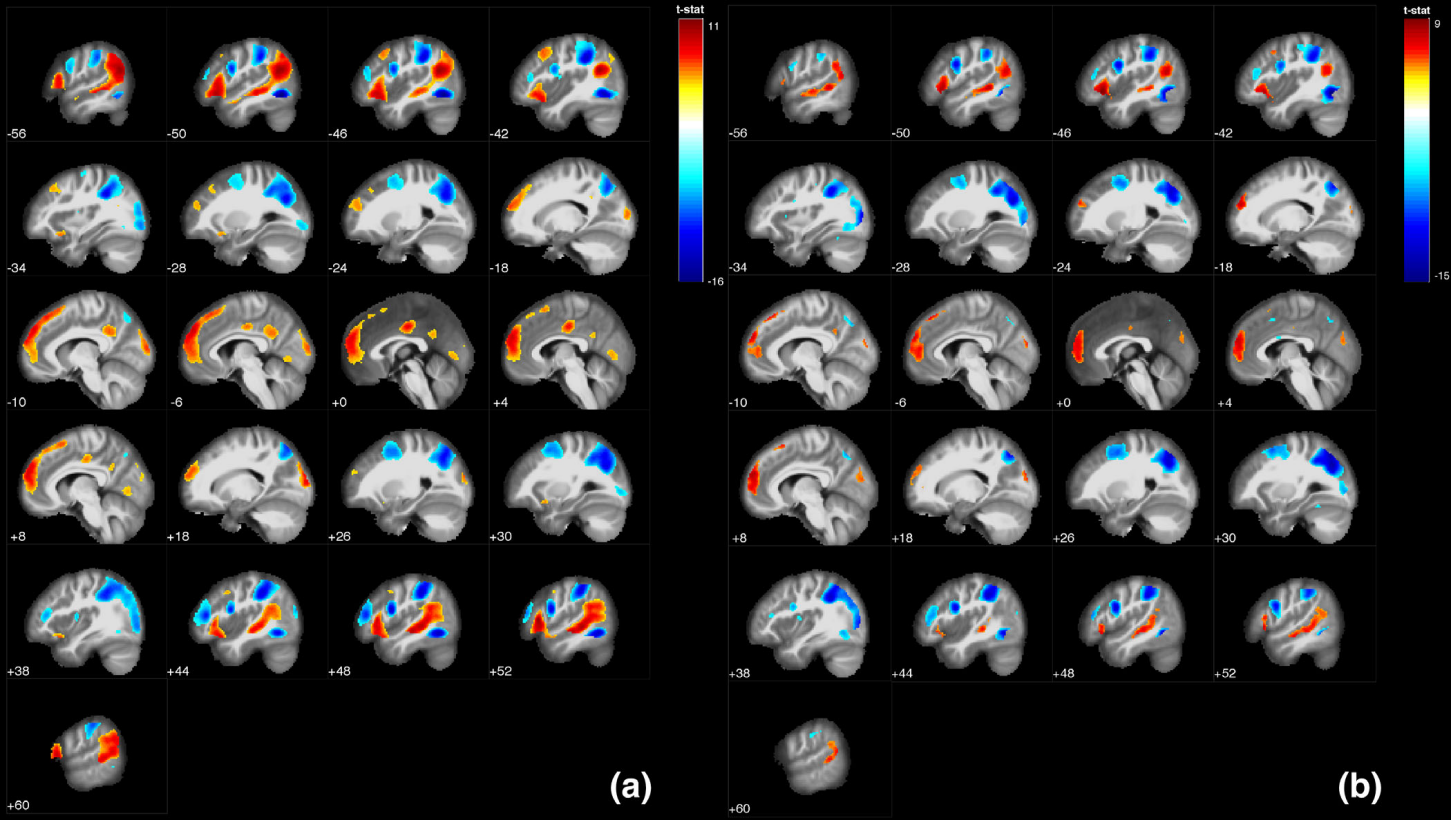
Replication Approach 1: Results from within‐sample, whole‐brain analyses of activation during each attention condition for the (a) California Families Project (CFP) sample (*N* = 156) and (b) Pittsburgh Girls Study of Emotion (PGS‐E) sample (*N* = 119). Regions activated during sadness introspection are shown in red‐yellow. Regions activated when judging a nonemotional facial feature (i.e., nose width) are shown in blue. Height threshold *t* > 4.70, *p* < .0000, family wise error corrected at *p* < 0.05; CFP *df* = 1,085, PGS‐E *df* = 826

As presented in Table 2, the flexible factorial whole‐brain analysis revealed a main effect of attention within multiple regions, a main effect of facial emotion in the fusiform gyrus (not shown), and a significant attention × facial emotion interaction in the PFC. A post hoc *t* test between attention conditions showed that engaging in introspection activated a social–emotional network comprising bilateral insula, lateral temporal regions, and portions of the mPFC and precuneus (Table 2; shown in red‐yellow in Figure 2, Panel a). In contrast, the nonemotional judgment condition activated bilateral dorsolateral and ventrolateral PFC as well as occipitotemporal regions (Table 2; shown in blue in Figure 2, Panel a). Neither cognitive reappraisal nor expressive suppression scores were associated significantly with whole‐brain activation.

**Table 2:** Activation foci for the interaction effect of face emotion × attention condition and each main effect of attention condition in the CFP sample (*N* = 156)

Brain region	Side	*x*	*y*	*z*	Cluster size	Statistic
Interaction effect: Face emotion × attention condition						
Medial prefrontal cortex	L	−6	24	40	30	28.36
Inferior frontal gyrus	R	54	26	24	15	26.02
Medial prefrontal cortex	L	−6	16	50	25	25.96
Main effect: Sadness introspection > nonemotional judgment						
Inferior frontal gyrus (pars triangularis)/temporal pole/medial temporal	R	56	24	6	1,159	11.68
MTG/inferior parietal lobule	L	−48	−60	24	3,115	10.96
MTG	R	50	−34	−2	2,909	10.46
Inferior frontal gyrus (pars orbitalis and pars triangularis)	L	−50	24	−8	1,313	10.19
Superior medial gyrus/superior frontal gyrus	R/L	6	60	26	5,254	9.63
Calcarine gyrus/cuneus/superior occipital gyrus	R	14	−98	12	577	9.05
Middle cingulate cortex		0	−16	40	497	8.45
Superior occipital gyrus/cuneus	L	−12	−98	12	493	7.55
PCC	R/L	−12	−46	34	579	6.93
Cerebellar cermis (6)		2	−76	0	289	5.77
Inferior frontal gyrus (pars opercularis)//precentral gyrus	R	44	10	44	53	5.39
Main effect: Nonemotional judgment > sadness introspection						
ITG/anterior intraparietal sulcus	L	−50	−64	−10	5,462	16.15
ITG	R	50	−56	−12	549	13.5
Supramarginal gyrus/anterior intraparietal sulcus/angular gyrus	R	50	−34	46	4,847	12.91
Inferior frontal gyrus (pars opercularis)	R	48	6	22	494	12.89
Middle frontal gyrus	R	46	38	14	777	10.48
Precentral gyrus/insula	L	−48	2	26	589	10.37
Middle frontal gyrus	R	28	8	54	786	8.58
Inferior frontal gyrus (pars triangularis)	L	−50	38	22	196	7.35
Middle frontal gyrus/precentral gyrus	L	−24	2	56	525	6.86
Insula lobe	R	38	−4	12	35	6.39

*Note*. Height threshold for the interaction effect was 22.40, *p* < .0142 and for the main effects was 4.70, *p* < .0000; *df* = 1,085; FWE corrected, *p* < .05; L, left; R, right.

Abbreviations: CFP, California Families Project; FWE, family wise error; ITG, inferior temporal gyrus; MTG, middle temporal gyri; PCC, posterior cingulate cortex.

**Figure 3:**
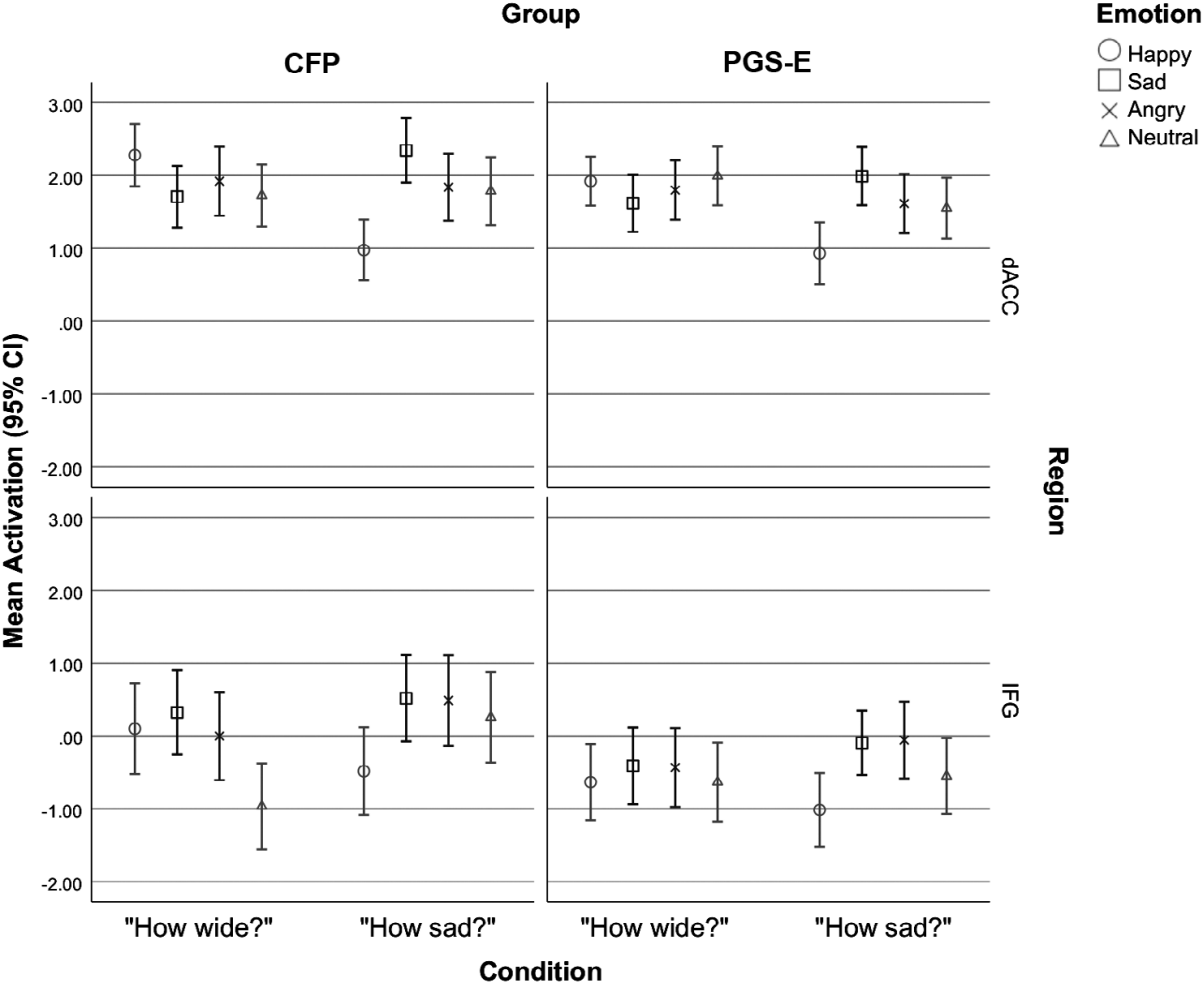
Replication Approach 1: Visualization of extracted mean beta parameters from the significant facial emotion × attention condition interaction effect (*F*[1,1,085] = 22.44, *p* < .014) in the dorsal anterior cingulate cortex (dACC; −6, 24, 40) and the right inferior frontal gyrus (IFG; 54, 26, 24) found in the whole‐brain analysis using the California Families Project (CFP) sample (*N* = 156). This interaction effect was not significant in the whole‐brain analysis using the Pittsburgh Girls Study of Emotion (PGS‐E) sample (*N* = 119), but the beta parameters are depicted here for visual comparison to the CFP sample

The attention × facial emotion interaction effect was concentrated to two small clusters (Table 2): one in the dorsal anterior cingulate cortex (dACC) and one in the right inferior frontal cortex (IFG). To identify what drove the interaction, we extracted beta parameters from these two clusters and plotted the interaction for visualization purposes. Figure 3 (CFP in left panel) shows the interaction effect was driven by opposing patterns of activation to sad and happy facial expressions in the two attention conditions.

We separately tested whether any of the effects differed between male and female participants, but did not find gender differences.

#### 3.1.2 PGS‐E sample

As shown in Table 3, the flexible factorial analysis at the whole‐brain level showed a significant main effect of attention. Similar regions were activated in the PGS‐E sample as seen in the CFP sample during sadness introspection (shown in red‐yellow in Figure 2, Panel b) and when making nonemotional judgments (shown in blue in Figure 2, Panel b). A main effect of facial emotion and an interaction effect of attention × facial emotion were not significant. Neither cognitive reappraisal nor expressive suppression scores were associated significantly with whole‐brain activation.

**Table 3:** Activation foci for each main effect of attention condition in the PGS‐E sample (*N* = 119)

Brain region	Side	*x*	*y*	*z*	Cluster size	Statistic
Main effect: Sadness introspection > Nonemotional judgment						
Inferior frontal gyrus (pars orbitalis)/temporal pole	L	−46	24	−6	514	9.41
Superior medial gyrus/superior frontal gyrus	R/L	6	60	22	2000	8.28
MTG	L	−50	−42	0	1,231	8.18
Inferior frontal gyrus (pars orbitalis, pars triangularis)	R	48	26	−8	128	7.48
Superior medial gyrus	L	−6	38	52	110	7.11
Insula/MTG	R	46	−32	−2	856	7.09
Calcarine gyrus/superior occipital gyrus	L	−8	−92	18	338	6.39
Posterior‐medial frontal/superior frontal gyrus/superior medial gyrus	R	8	18	62	80	6.21
Middle frontal gyrus	L	−40	10	48	46	6.19
PCC	L	−10	−50	34	16	5.32
Premotor cortex	L	−10	20	60	11	5.32
Premotor cortex		0	−14	38	19	5.01
Main effect: Nonemotional judgment > sadness introspection						
Fusiform gyrus/superior parietal lobule/middle occipital Gyrus	L	−42	−68	−8	3,791	15.65
Superior parietal lobule/angular gyrus/supramarginal gyrus	R	30	−68	36	3,560	13.82
Inferior frontal gyrus (pars opercularis)	R	48	6	26	547	12.32
Inferior frontal gyrus (pars opercularis)	L	−48	4	30	517	11.75
ITG	R	44	−62	−8	295	11.69
Middle frontal gyrus/premotor cortex	R	26	4	52	738	8.64
Middle frontal gyrus	L	−24	2	52	429	8.26
Middle frontal gyrus/inferior frontal gyrus (pars triangularis)	R	48	38	16	280	7.96
Inferior frontal gyrus (pars triangularis)	L	−46	36	18	121	6.31
Insula lobe	R	38	−4	12	18	5.65
Cerebellum (VI)	R	30	−50	−20	18	5.58
Insula lobe	L	−32	14	6	19	5.55
Premotor cortex		4	4	24	11	5.22
Middle cingulate cortex		4	14	48	17	5.20

*Note*. Height threshold for the main effects was 4.70, *p* < .0000; *df* = 826; FWE corrected, *p* < .05; L, left; R, right.

Abbreviations: FWE, family wise error; ITG, inferior temporal gyrus; MTG, middle temporal gyri; PCC, posterior cingulate cortex; PGS‐E, Pittsburgh Girls Study of Emotion.

Although we did not find a significant attention × facial emotion interaction effect in the PGS‐E sample, we examined whether the dACC and right IFG clusters observed in the CFP sample would show a similar pattern in the PGS‐E sample. To do so, we extracted mean beta parameters from the same clusters that were significant in the CFP sample in the PGS‐E sample. As shown in Figure 3 (PGS‐E in right panel), the visualization of the pattern of activation in the PGS‐E sample parallels the one observed in the CFP sample overall, including the differences in activation to happy and sad faces in the two attention conditions.

#### 3.1.3 Conjunction analysis

**Figure 4:**
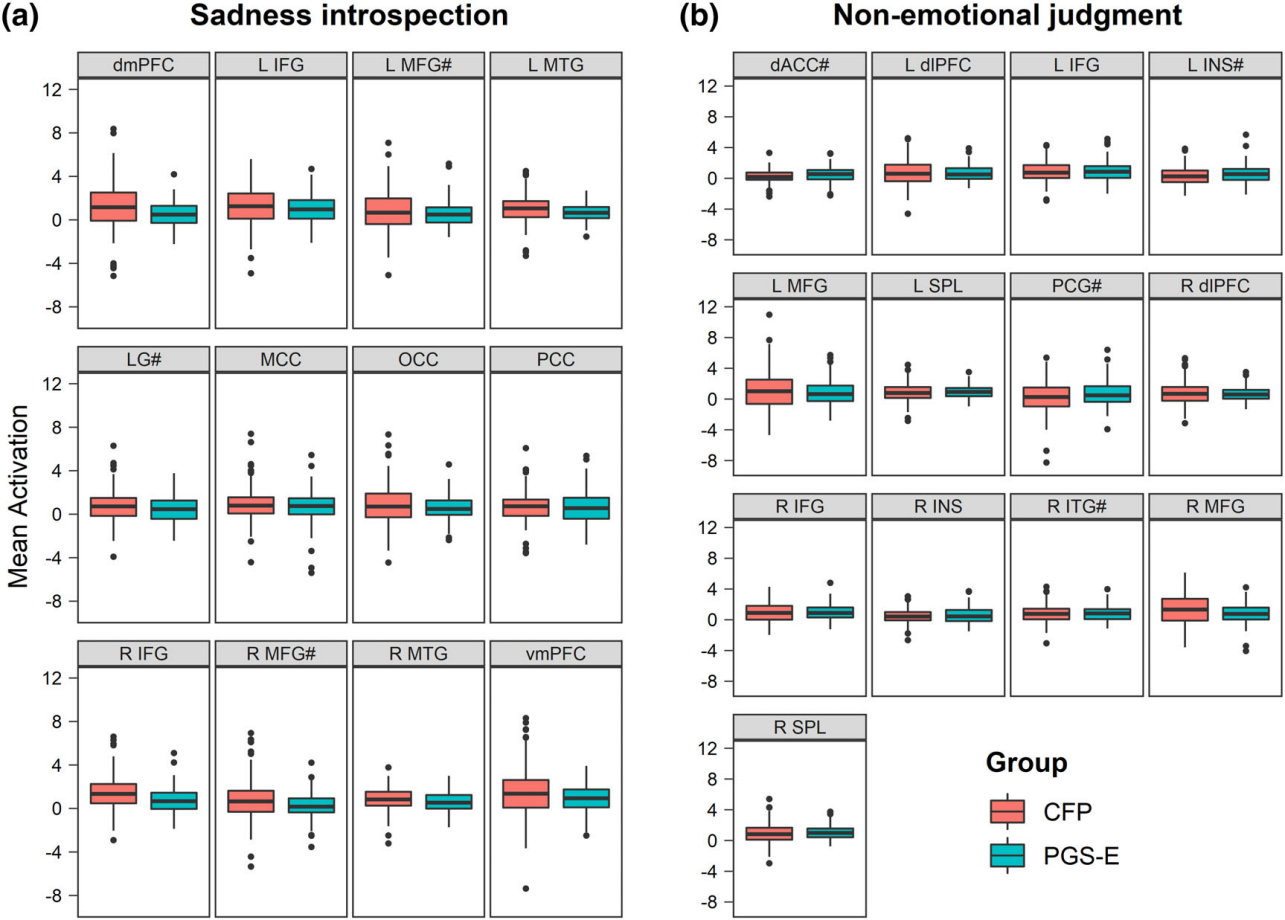
Replication Approach 1: Results from the conjunction map analysis of each cluster significantly activated in both samples for the sadness introspection and nonemotional judgment contrasts for the California Families Project (CFP) sample (*N* = 156) shown in red and the Pittsburgh Girls Study of Emotion (PGS‐E) sample (*N* = 119) shown in green. Boxplots depict mean activation for overlapping clusters unless otherwise indicated by #. dACC, dorsal anterior cingulate cortex; dlPFC, dorsolateral prefrontal cortex; dmPFC, dorsomedial prefrontal cortex; IFG, inferior frontal gyrus; INS, insula; ITG, inferior temporal gyrus; L, left; LG, lingual gyrus; MCC, midcingulate cortex; MFG, middle frontal gyrus; MTG, medial temporal gyrus; OCC, occipital pole; PCC, posterior cingulate cortex; PCG, paracingulate gyrus; R, right; SPL, superior parietal lobule; vmPFC, ventromedial prefrontal cortex

Based on a conjunction map of regions activated in both samples, Figure 4 shows the distribution of mean activation within each sample for each conjunct cluster (CFP in red; PGS‐E in green). For both samples, as depicted in the boxplots, there was more variance in the introspection than the nonemotional judgment attention condition. Notably, there were three clusters with significant levels of activation in only one of the samples while engaged in sadness introspection; the left middle frontal gyrus (MFG) (−40, 10, 48; PGS‐E only), medial lingual gyrus (2, −76, 0; CFP only), and right MFG (44, 10, 44; CFP only). When making a nonemotional judgment, four clusters did not overlap between samples including the dACC (4, 4, 24; PGS‐E only), left insula (−32, 14, 6; PGS‐E only), paracingulate gyrus (4, 14, 48; PGS‐E only), and right ITG (50, −56, −12; CFP only).

### 3.2 Replication Approach 2: Between sample differences across the whole brain within the same model

A two‐sample *t* test revealed two small clusters of greater activation in the CFP sample compared to the PGS‐E sample during emotion introspection (*p* < .05, FWE‐corrected). These activation clusters were in the right IFG (54, 20, 0; *k* = 20) and the mPFC (−8, 54, 36; *k* = 14), but were no longer significantly different between samples when gender and expressive suppression scores were entered as covariates. When examining the effects of gender at the whole‐brain level across the two samples, no significant activations were found.

### 3.3 Replication Approach 3: Between sample differences in signal change from a priori defined ROIs

**Figure 5:**
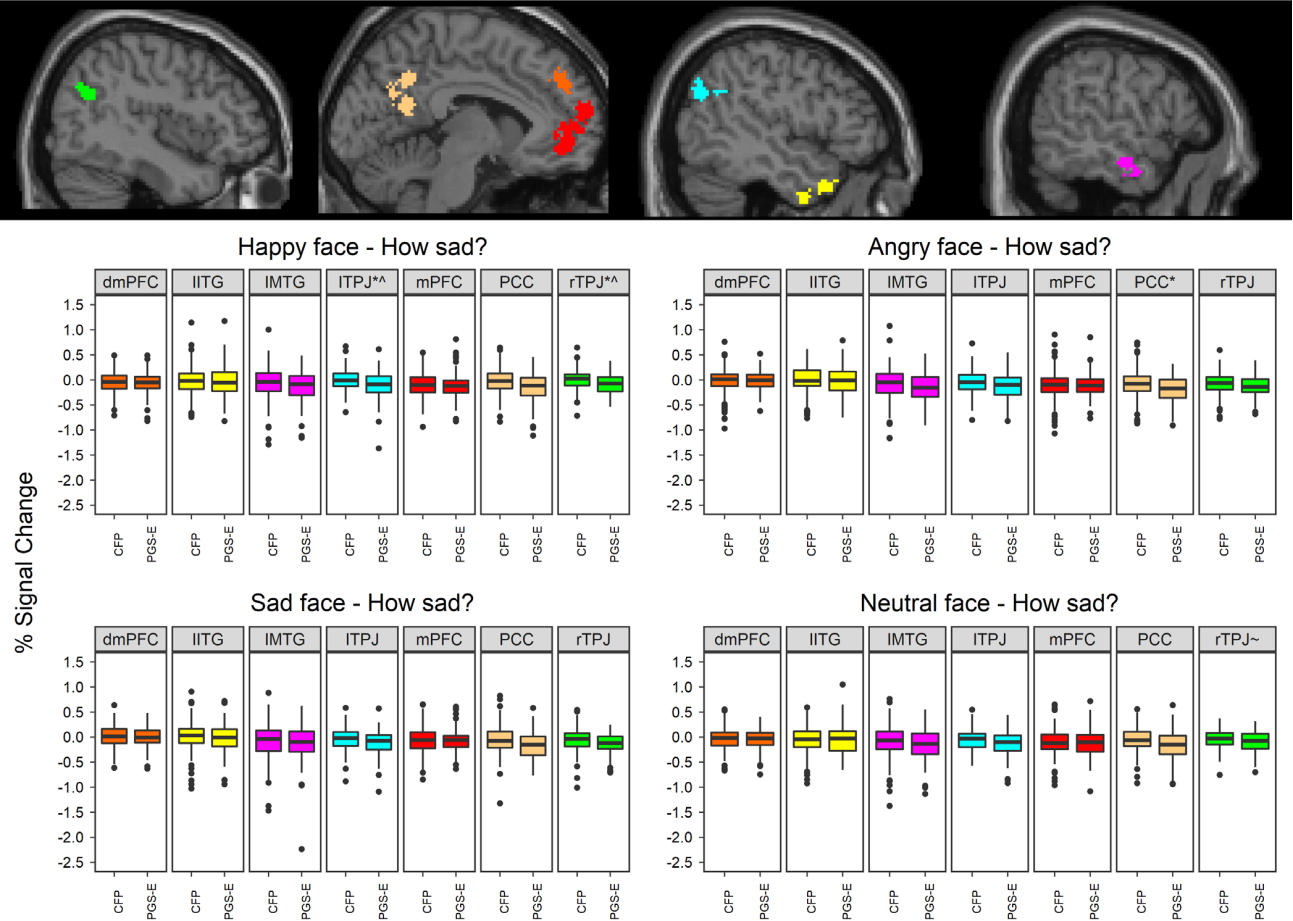
Replication Approach 3: The top panel shows seven a priori defined regions of interest (ROIs) based on the term “self‐referential” from the Neurosynth database. ROIs are the dorsomedial prefrontal cortex (dmPFC, dark blue), left inferior temporal gyrus (lITG, purple), left middle temporal gyrus (lMTG, yellow), left temporoparietal junction (lTPJ, light blue), medial prefrontal cortex (mPFC, red), posterior cingulate cortex (PCC, beige), and right temporoparietal junction (rTPJ, green). The bottom panel displays the percent signal change within each ROI for each of the four facial emotions viewed during sadness introspection (“How sad does this face make you feel?”). CFP, California Families Project sample (*N* = 156). PGS‐E, Pittsburgh Girls Study of Emotion sample (*N* = 119). *Significant at *p* < .0018 (Bonferroni corrected); ^remained significant after covarying gender and expressive suppression; ~became significant at *p* < .0018 after covarying gender and expressive suppression

To further probe replicability of task activations across the two samples, we used seven a priori defined ROIs from the Neurosynth database based on the term “self‐referential” to select regions commonly activated by introspective task conditions (see Figure 5, top panel). Mean percent signal change for each group, each ROI, and each facial expression during sadness introspection are presented in the bottom panel of Figure 5 and Table S3. Four of the ROIs including the mPFC, dmPFC, left MTG, and left ITG showed no significant differences in percent signal change between the groups. Accounting for multiple comparisons, percent signal change was significantly different for happy facial expressions only in the left and right TPJ and for happy and angry expressions in the PCC. For the significant regions, the PGS‐E sample exhibited overall greater negative signal change compared to the CFP sample. When both gender and expressive suppression scores were included as covariates, group differences remained significant for happy facial expressions in bilateral TPJ and in the PCC. Angry facial expressions in the PCC no longer showed group differences, instead response to neutral facial expressions in the left TPJ was found to differ significantly (*p* = .0012) between the groups after entering the covariates. However, no significant effects of expressive suppression scores or gender were found in any of the models tested.

## 4 DISCUSSION

In the present study, we characterized and directly replicated whole‐brain and ROI neural activation patterns of sadness introspection in two independent samples of adolescents with a variant of a widely used facial emotion processing fMRI task. We examined replication using three different approaches. Across these approaches, activation patterns were comparable between the samples during sadness introspection. When adolescents focused their attention on the degree of sadness they felt while looking at emotional faces, we found activation in the DMN and other regions typically implicated in social cognition, emotion processing, and self‐referential processing. This included bilateral middle temporal gyri, inferior and middle frontal gyri, cortical midline regions including the PCC, mPFC, and midcingulate as well as visual processing regions within the occipital cortex. When adolescents made nonemotional judgments, that is, rated a face's nose width, we found activation in regions corresponding to a dorsal attention network including the supplementary motor region, superior parietal cortices, insula, and inferior and dorsolateral prefrontal regions in both samples. Overall, the similar patterns of activation found between the two independent samples suggest broad replicability of results. They also support the reliability of this task for engaging the same brain regions in response to specific social cognitive events in different groups of adolescents.

In support of cross‐sample replication of neural activation, our first replication approach demonstrated extensive overlap in clusters that were significant within each sample. When activation in certain clusters was significant in only one sample, we extracted the BOLD signal from the other sample using masks defined by those same clusters including bilateral middle frontal gyri, the lingual gyrus, dACC, left insula, and right IFG and found no significant mean activation differences between the samples. In addition, although the interaction effect of what participants rated (i.e., attention condition) and the type of facial emotion they rated was significant only in the CFP sample at the whole‐brain level, the same general response pattern was also observed in the PGS‐E sample in these regions. Overall, the results from this approach suggest the facial emotion processing fMRI task reliably elicits activation patterns across research sites and across two diverse samples of adolescents. Furthermore, the findings indicate the instructions used in social–emotional cognitive tasks can effectively manipulate neural response even when task stimuli are identical across conditions. The way in which adolescents process facial emotions—whether appraising them in relation to their own subjective emotional experience or based on a nonemotional feature of the face—influences the neural representation and engagement of the perceived emotion.

We undertook two additional analytic approaches for assessing replication of one sample's results in another sample. Our second replication approach, in which we directly compared the two samples in one analytic model, showed no neural activation differences between the samples including in those regions that were significantly activated in only one sample (as found in replication approach one). The second replication approach did reveal significant between‐sample differences in a small cluster of activation in the right IFG and one in the mPFC. However, when gender was included as a covariate, these differences were no longer significant. Nonetheless, when we examined the effect of gender at the whole‐brain level no significant associations were found. Additionally, expressive suppression was not associated with activation during introspection. Results from this second replication approach lend further support to the findings obtained from the first approach and revealed some neural activation differences that may have been related to the divergence in gender composition of each sample.

For the third replication approach, we created ROIs involved in introspection based on the literature. This approach measured the percent signal change in a priori defined regions, including mPFC, PCC, left MTG, left TPJ, dmPFC, left ITG, and right TPJ. Across the majority of ROIs, no significant differences in activation during introspection were found between the samples. However, differences were noted in bilateral TPJ for happy facial expressions and in the PCC for happy and angry expressions; most of these associations remained significant when controlling for between sample differences in gender and expressive suppression. These results indicated that the two samples generally processed task conditions similarly within a network of regions identified in past work as relevant for engaging in introspection and self‐referential processes (e.g., the DMN).

We also explored whether self‐reported emotion regulation skills assessed in each sample using the same measure (i.e., ERQ) would be associated with task‐based neural activations. We found no evidence for an effect of emotion regulation at the whole‐brain level in either sample (replication approach one). While this is not evidence of no effect, it has been noted that brain–behavior correlations show low replicability (albeit in structural studies, see Boekel et al., 2015) and are often based on weak, false, or hidden correlations (Rousselet & Pernet, 2012). In addition, the subscales of the ERQ, cognitive reappraisal and expressive suppression, may be too broad as constructs to map onto activation of any specific brain region(s), especially given the current task design. In the context of Gross's model of emotion regulation (Gross, 2008, 2015), the current task relates most closely to attention deployment, which precedes both reappraisal and suppression of one's emotions. Furthermore, sadness introspection as operationalized with this task captures neural response when thinking about how another person's emotional display makes you feel sadness, rather than asking participants explicitly to change their current emotion state by reframing it or suppressing it.

In addition to the results obtained from our replication efforts, the current study revealed intriguing patterns about neural engagement during sadness introspection. First, the conjunction analysis showed there was more variance in neural activation during introspection as compared to the nonemotional judgment condition in both samples. It is possible that this pattern reflected greater individual differences in neural activity during sadness introspection. The greater idiosyncratic reaction to one's own emotional response is perhaps more influenced by past experiences or current mood than when rating a nonemotional feature. In contrast, we would expect activity when making a nonemotional judgment to be more similar across individuals.

Second, the dACC and right IFG showed differential activation to happy and sad faces depending on whether participants were engaged in introspection or a nonemotional judgment. The dACC and IFG are both prominent cognitive control regions engaged during error monitoring, implicit and explicit emotion regulation, response inhibition and general attention monitoring (e.g., Chambers, Garavan, & Bellgrove, 2009; Luna, Padmanabhan, & O'Hearn, 2010; Ochsner, Silvers, & Buhle, 2012). The dACC also falls within the salience network, which is involved in integrating internal events and environmental stimuli (Menon & Uddin, 2010) theorized to mediate switching between the DMN and a central executive network and be implicated in psychopathology (Menon, 2015). In the context of this task and given involvement of the dACC during error monitoring and conflict detection (Botvinick, Cohen, & Carter, 2004), the interaction we found is likely driven by a mismatch between viewing a happy face but considering one's sad feelings during introspection. In addition, the nonemotional judgment condition, which inherently activates implicit processing of emotions, may require participants to increase their attention when viewing sad faces in order to stay on task. Such an effect may be associated with a need to remind oneself of the current condition and not interfere with thinking about one's feelings of sadness.

Third, the significant differences in dmPFC and right IFG activation during introspection found between the two samples did not hold when gender was included as a covariate the model, suggesting that gender may have played a role in these differences. Both dmPFC and right IFG engagement have been associated with cognitive and inhibitory control. In the context of the present task, the dmPFC was likely involved in emotion regulation and awareness (Amodio & Frith, 2006) as well as changes in affective experience (Silvers, Wager, Weber, & Ochsner, 2015). Gender differences in brain activity associated with emotional reactivity and regulation have been observed in adults (Domes et al., 2010). Adult women show greater amygdala, mPFC, and dorsolateral PFC activity compared to men when viewing aversive stimuli. In contrast, when instructed to downregulate an emotional response to negative stimuli, men showed greater engagement in the caudal ACC, lateral OFC, and inferior PFC compared to women (Domes et al., 2010). Together, these observations suggest the neural activation differences during sadness introspection observed in our second replication approach may have been related to differences in the gender composition of each sample. However, it is possible that other variables that may have accounted for these differences (e.g., research site, MRI acquisition parameters, cultural, social, and/or socioeconomic influences) because when we tested for the effect of gender, we did not find significant associations between gender and neural activation at the whole‐brain level.

Neuroimaging research has been criticized for solely relying on statistical values in the absence of a direct physical measurement (Chen, Taylor, & Cox, 2017). Because reporting effect sizes in fMRI is challenging, we included a third replication approach that assessed percent signal change, a metric proposed to be as close to an effect size as is currently possible (Chen et al., 2017). Our results from this analysis showed that when comparing percent signal change in a priori defined ROIs, there were differences between the two samples in the bilateral TPJ and PCC for happy facial expressions, including when accounting for gender and expressive suppression effects. Engaging in mentalizing processes, such as representing one's own thoughts and emotions or the thoughts and emotions of another person implicates the TPJ and PCC (Lombardo, et al., 2010; Blakemore, 2008). However, the TPJ also supports attentional processes such as reorientation to behaviorally important stimuli in the environment (Arrington, Carr, Mayer, & Rao, 2000; Corbetta, Patel, & Shulman, 2008). Together with the PCC, the TPJ overlaps with lateral parietal regions within the DMN, which deactivates during effortful tasks. Previous work indicates that DMN deactivation is less pronounced for happy faces as compared to sad faces (Sreenivas et al., 2012). Group differences seen specifically for happy faces may thus indicate differences in attention orientation or DMN suppression.

Activation in the TPJ and PCC has been associated with individual differences in loneliness (e.g., Cacioppo & Hawkley, 2009) as well as empathy and the ability to infer social intentions (e.g., Ciaramidaro et al., 2007). In addition, reduced suppression of DMN activity during affective processing has been reported in depressed adults (Grimm et al., 2009) and adolescents (Ho et al., 2015) with PCC activation correlating positively with feelings of depression and hopelessness (Grimm et al., 2009) and PCC connectivity correlating with greater depression severity and an earlier age of depression onset (Ho et al., 2015). Although our two samples were recruited into studies that assessed risk for depression, they were drawn from the community and are not comparable to clinical samples. Nevertheless, it is possible that individual differences between the samples or greater psychopathology in one or the other sample may account for the observed differences in signal change.

An alternative explanation for group differences in the processing of happy facial expressions may also be due to the specific set of task stimuli used. Facial expression stimuli were not explicitly selected to match each sample's or each participant's race/ethnicity. In the present study, we used these samples as a way to highlight a quantitative difference in the brain regions activated during introspection and to show that this task is appropriate to use in different communities. As a post hoc examination of potential group differences between race/ethnicity, we compared mean percent signal change in bilateral TPJ and PCC response to happy facial expressions across Mexican‐origin, African‐American, and non‐Hispanic White participants. Consistent with our site/sample differences, Mexican‐origin (CFP) and African‐American (PGS‐E) and Mexican‐origin (CFP) and non‐Hispanic White (PGS‐E) participants differed in TPJ and PCC activity to happy facial expressions, but African‐American (PGS‐E) and non‐Hispanic White (PGS‐E) participants did not differ (*p*‐values = .10–.95). Thus, within the PGS‐E sample, there were no ethnic/racial differences in neural activity. Nonetheless, our ability to test alternative explanations related to group differences between race/ethnicity with these datasets is limited because race/ethnicity is confounded with site/sample. Future studies might address the question of how race or ethnicity relates to TPJ and PCC responses to varying facial emotions during introspection by accounting for the cultural context in which emotion socialization took place and systematically controlling for the race/ethnicity of the facial stimuli.

Our study was not without some limitations. The replication of activation patterns for each attention condition we found across samples is remarkable, although not perfect. First, significant clusters were more extensive in the CFP sample suggesting that a slightly larger sample size may influence the ability to detect significant effects. Given that the interaction effect was nearly identical in both samples, although only significant in one sample, judging replication solely based on a significant cluster cutoff may have been too stringent. Although there is close overlap in activations between the samples, differences found in the order of cluster significance also supports this possibility. Thus, only relying on clusters that pass a certain *t*‐statistic threshold may not be a good indicator of replication, since we found no group differences in clusters that reached significance in only one group. Second, we tested whether mean activation in ROI clusters differed across the samples; however, future work could apply multivariate methods (e.g., multivoxel pattern analysis) to address new questions about similarity of neural response patterns between samples. Third, our study should be considered exploratory given the absence of guidelines and clear methodological approaches to test replicability in neuroimaging research. As neuroimaging data are increasingly made publicly available, neuroimaging researchers will likely develop gold‐standard approaches for assessing replication. In addition, the ROIs we selected based on the Neurosynth results were generated from a range of studies that included participants of all ages. While Neurosynth provides no information on the age of each sample used in their meta‐analysis algorithm, it is highly likely that more of the studies drew on adult than adolescent samples. Nonetheless, our whole‐brain and ROI replication approaches showed engagement of similar regions, supporting the validity of using Neurosynth‐based ROIs for the age of our sample. Fourth, the design of our fMRI task was low in ecological validity. Indeed, we designed the task to experimentally manipulate attention and assess response to emotional stimuli, but it does not represent the complex social–emotional processes involved in adolescents' naturalistic interpersonal interactions. Future studies should aim to use paradigms that more accurately reflect the social and emotional experiences adolescents encounter in daily life (Guyer et al., 2016). Finally, although leveraging datasets from two separate studies provided an important opportunity to replicate findings from an fMRI task collected from large samples of same‐aged adolescents, our study design was limited by the unique demographic breakdown of the samples, slight differences in functional and structural imaging parameters (e.g., flip angle, FOV), and differing behavioral/clinical measures. For example, the somewhat smaller FOV of the functional T2‐weighted echo planar images for the PGS‐E scan may have provided higher resolution and smaller voxel size, but lower signal strength than the FOV for the CFP scan, which may have created slight differences in activation patterns. In addition, each study collected different measures of depressive severity and rumination at different assessment times relative to the scan. This precluded our ability to compare the two samples directly on these measures and limits interpretation of any group differences in neural activation in relation to these psychological processes.

Taken together, the findings from the present multisite study suggest it is possible to replicate reliable and robust BOLD signal in core social–emotional and cognitive control processing regions during relevant task conditions. The ability to reflect upon one's own emotional state in the presence of another person's emotional expressions is a skill necessary for successful social interaction. The brain regions significantly activated when participants engaged in introspection and made nonemotional judgments are consistent with regions reported in previous studies engaged during social cognition tasks as well as emotional tasks (Schilbach et al., 2012). In the present study, we showed that a variation of a commonly used facial emotion face‐processing fMRI task largely replicated activation patterns across two independent adolescent samples. Furthermore, these results support the use of a facial emotion‐processing task in future neuroimaging studies bearing in mind that task instructions are important in driving region‐specific activation and variability in the BOLD signal.

## CONFLICT OF INTEREST

The authors declare no conflict of interest.

## Supporting information


**Appendix S1:** Supporting Information

## Data Availability

The data that support the findings of this study are available from the corresponding author upon reasonable request.
