# Supplementary material for: Direct replication of task‐dependent neural activation patterns during sadness introspection in two independent adolescent samples
Source: Hum Brain Mapp. 2019 Oct 22;41(3):739–54. doi: 10.1002/hbm.24836 (PMC6980880; doi:10.1002/hbm.24836)
Supplement: Supplementary file 1 — Appendix S1: Supporting Information [file HBM-41-739-s001.docx]

**Supplementary Material**

Figure S1. Overlap of clusters for the (A) sadness introspection and (B) non-emotional judgement conditions from the conjunction map analysis. Activation clusters are shown in blue for the California Families Project (CFP) sample (*N* = 156) and shown in green for the Pittsburgh Girls Study of Emotion (PGS-E) sample (*N* = 119).

| **A** | 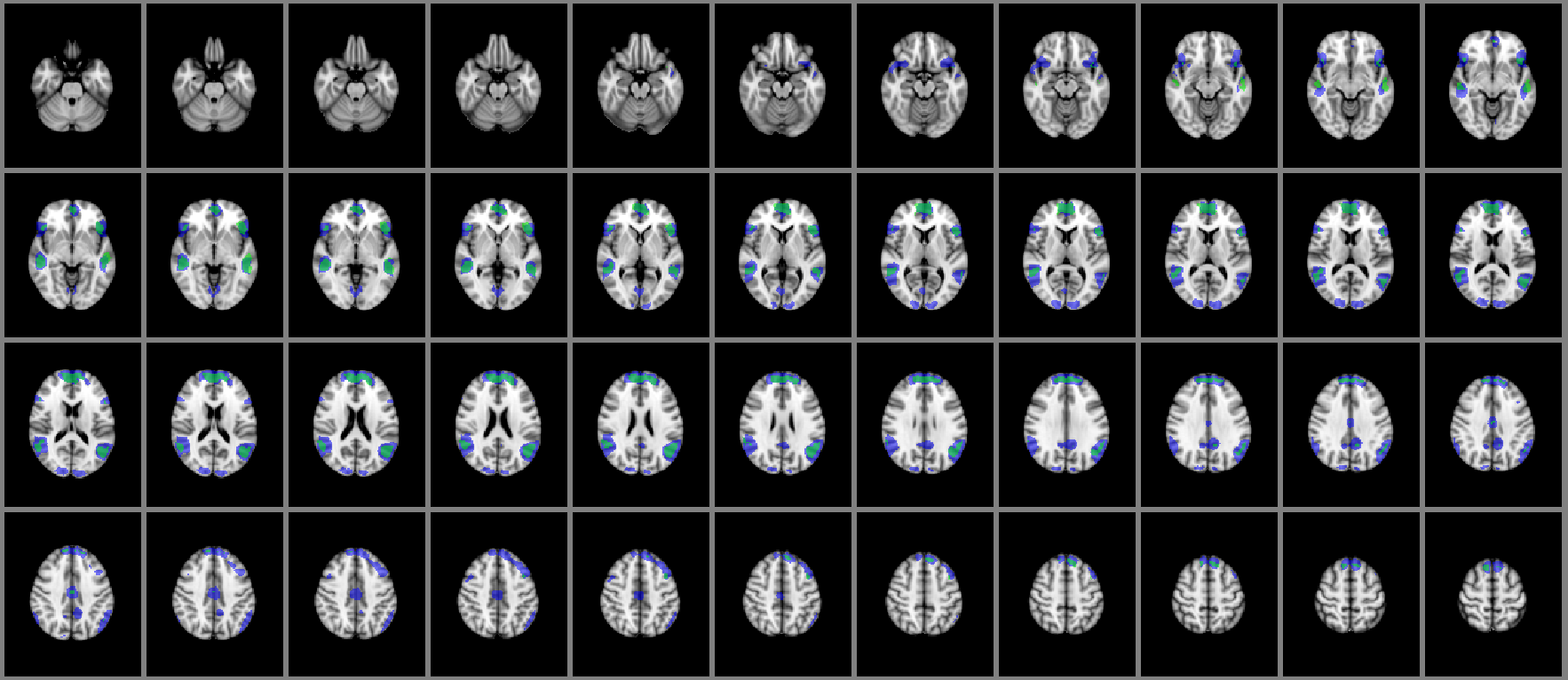 |
| --- | --- |
| **B** | 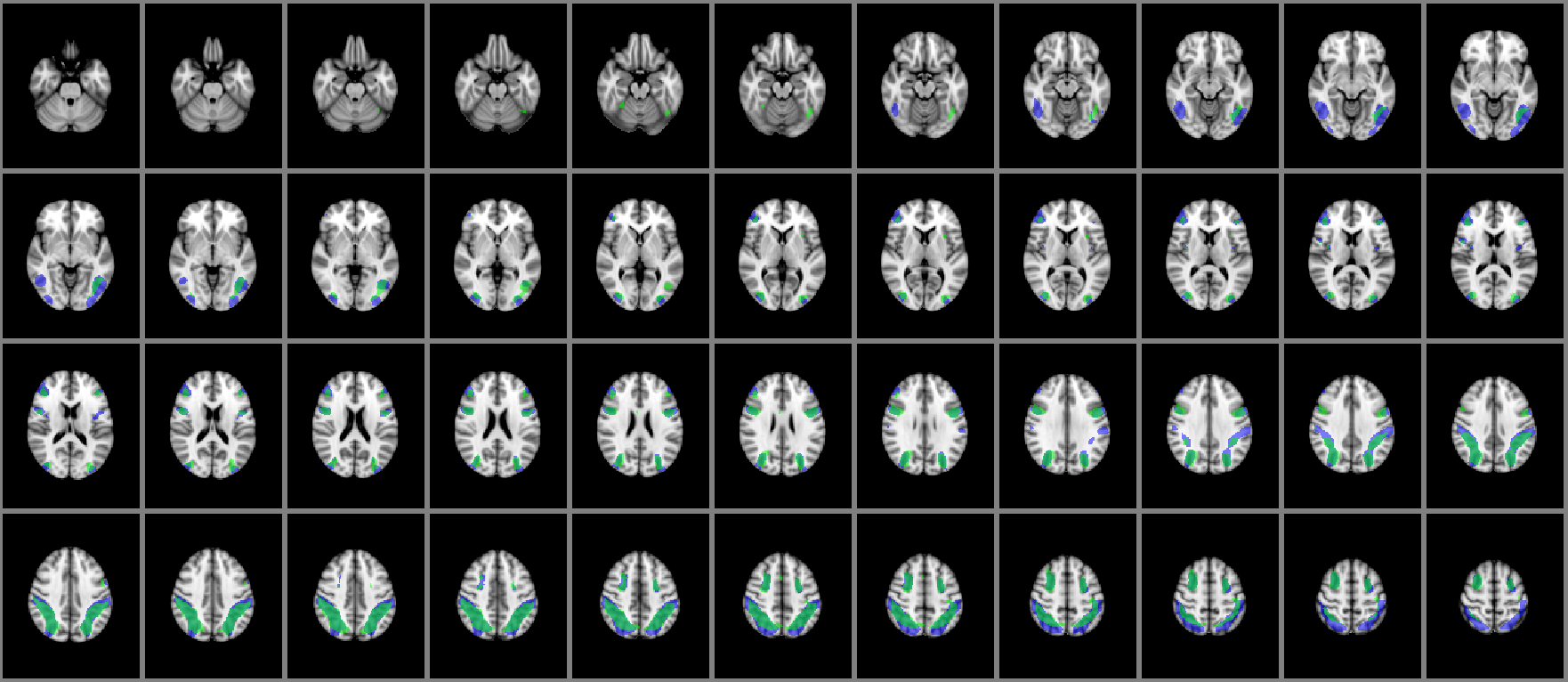 |

Table S1. Peak voxel coordinates for significant clusters in the California Families Project (CFP) sample (*N* = 156) and corresponding peak voxel coordinates in the Pittsburgh Girls Study of Emotion (PGS-E) sample (*N* = 119) that were combined to form an overlapping region-of-interest to probe for group differences during sadness introspection and non-emotional judgments.

| **CFP Cluster**  **(peak voxel coordinates)** | **Matching PGS-E Cluster**  **(peak voxel coordinates)** | **Label of Overlap** |
| --- | --- | --- |
| Introspection > Non-emotional judgement |  |  |
| 0 -16 40 | 0 -14 38 | middle cingulate |
| -12 -46 34 | -10 -50 34 | PCC |
| -50 24 -8 | -46 24 -6 | left IFG |
| 50 -34 -2 | 46 -32 -2 | right MTG |
| 56 24 6 | 48 26 -8 | right IFG |
| -48 -60 24 | -50 -42 0 | left MTG |
| 6 60 26 | -10 20 60 & -6 38 52 | dmPFC |
| 6 60 26 | 6 60 22 | vmPFC |
| 14 -98 12 & -12 -98 12 | -8 -92 18 | bilateral occipital cortex |
| 2 -76 0 | --- | lingual gyrus |
| 44 10 44 | --- | right MFG |
| --- | -40 10 48 | left MFG (BA 44) |
|  |  |  |
| Non-emotional judgement > Introspection |  |  |
| -24 2 56 | -24 2 52 | left dlPFC |
| 28 8 54 | 26 4 52 | right dlPFC |
| 50 -34 46 | 30 -68 36 | right SPL |
| 38 -4 12 | 38 -4 12 | right insula |
| -50 -64 -10 | -42 -68 -8 | left SPL |
| -50 38 22 | -46 36 18 | left MFG (BA 45) |
| 46 38 14 | 48 38 16 | right MFG (BA 45) |
| -48 2 26 | -48 4 30 | left precentral / IFG (BA 44) |
| 48 6 22 | 48 6 26 | right precentral / IFG (BA 44) |
| 50 -56 -12 | ---- | right inferior temporal gyrus |
| --- | -32 14 6 | left insula |
| --- | 4 14 48 | paracingulate gyrus |
| --- | 4 4 24 | dorsal ACC |

Note: PCC = posterior cingulate cortex; IFG = inferior frontal gyrus; MTG = middle temporal gyrus; dmPFC = dorsomedial prefrontal cortex; vmPFC = ventromedial prefrontal cortex; MFG = middle frontal gyrus; SPL = superior parietal lobe; ACC = anterior cingulate cortex; BA = Brodmann area

**Behavioral Performance in Each Sample**

Table S2 presents means and SDs for “How sad does this person make you feel?” and “How wide is the nose?” ratings and reaction times (RTs) per facial emotion within each sample.

**CFP sample*.*** Facial emotion had a significant effect on sadness introspection ratings, *F*(2.03,312.63) = 89.68, *p* < .001. Each facial expression was significantly differently rated from each of the other expressions (all *p* < .001; except for neutral and angry faces that were significantly different at *p* = .011). Sad facial expressions were rated as inducing the greatest feelings of sadness and happy facial expressions the least (Table S2). Facial emotion also had a significant effect on non-emotional judgment ratings, *F*(3,462) = 44.41, *p* < .001. Each facial expression was rated significantly different from each of the other expressions (all *p* < .001) with the exception of sad and neutral faces, which did not differ significantly in their average nose-width ratings (*p* = 1.0).

For RTs, there was a significant main effect of attention (i.e., introspection or non-emotional judgment), *F*(1,154) = 48.44, *p* < .001, and of emotion, *F*(2.77,426.74) = 52.10, *p* < .001, as well as a significant attention × emotion interaction effect, *F*(2.67, 411.61) = 73.72, *p* < .001 (Figure S2 depicts the interaction effect). The interaction effect indicated significantly faster RTs to happy faces versus neutral faces in the introspection compared to the non-emotional judgment condition, *F*(1,154) = 90.36, *p* < .001. Sad faces were responded to significantly more slowly compared to neutral faces in the introspection as compared to the non-emotional judgment condition, *F*(1,154) = 7.92, *p* = .008. RTs to angry faces (compared to neutral) did not differ between the two conditions, *F*(1,154) = .60, *p* = .448.

**PGS-E sample.** As shown in Table S2, facial emotion had a significant effect on sadness introspection ratings, *F*(2.00,236.42) = 105.19, *p* < .001, with each facial expression rated significantly different from each of the other expressions (all *p* < .005). Sad facial expressions were rated as inducing the greatest feelings of sadness and happy facial expressions the least (Table S2). Facial emotion also had a significant effect on non-emotional judgment ratings, *F*(2.78,327.73) = 73.40, *p* < .001. When making non-emotional judgments, each facial expression was rated significantly different from each of the other expressions (all *p* < .005).

For RTs, there was a significant main effect of attention, *F*(1,118) = 39.57, *p* < .001, and emotion, *F*(2.66,313.73) = 38.68, *p* < .001, and a significant attention × emotion interaction effect, *F*(3,354) = 40.88, *p* < .001. The interaction effect was driven by significantly faster RTs to happy versus neutral faces during the sadness introspection compared to the non-emotional judgment condition, *F*(1,118) = 69.49, *p* < .001 (also see Figure S2). RTs to sad and angry faces (versus neutral) did not differ between the introspection and non-emotional judgement conditions, *F*(1,118) = .60, *p* = .442 and *F*(1,118) = 1.97, *p* =.163, respectively.

Overall, both samples showed similar patterns of engagement with the task based on their subjective ratings and RTs. Both samples rated sad faces as inducing the greatest feelings of sadness and happy faces as the least. However, the CFP sample rated nose width of sad and neutral faces similarly whereas the PGS-E sample rated them significantly different in nose width. RTs varied as a function of both facial emotion and attention in both samples, as evidenced by the significant interaction effects found in both samples. Both samples showed significantly faster RTs to happy versus neutral faces when rating their subjective feelings of sadness versus when rating a non-emotional facial feature.

Table S2. Mean (SD) ratings and reaction times (RT) for each face emotion (happy, sad, angry, neutral) and attention condition (introspection: How sad does this face make you feel?, non-emotional judgment: How wide is the nose?)

|  | **Happy** | | **Sad** | | **Angry** | | **Neutral** | |
| --- | --- | --- | --- | --- | --- | --- | --- | --- |
|  | ***M*** | ***SD*** | ***M*** | ***SD*** | ***M*** | ***SD*** | ***M*** | ***SD*** |
| **CFP (*n* = 156)** |  |  |  |  |  |  |  |  |
| Rating: How sad? | 1.30 | 0.66 | 2.20 | 0.87 | 1.78 | 0.75 | 1.66 | 0.65 |
| Rating: How wide? | 2.51 | 0.44 | 2.17 | 0.45 | 2.38 | 0.46 | 2.21 | 0.40 |
| RT: How sad? | 1199.70 | 243.46 | 1515.01 | 319.45 | 1471.81 | 365.43 | 1437.38 | 346.76 |
| RT: How wide? | 1548.09 | 267.76 | 1508.55 | 235.31 | 1544.70 | 262.19 | 1492.45 | 250.07 |
| **PGS-E (*n*=119)** |  |  |  |  |  |  |  |  |
| Rating: How sad? | 1.32 | 0.56 | 2.67 | 1.11 | 2.00 | 0.99 | 1.79 | 0.72 |
| Rating: How wide? | 3.15 | 0.64 | 2.51 | 0.53 | 2.88 | 0.62 | 2.64 | 0.54 |
| RT: How sad? | 1248.95 | 247.46 | 1486.39 | 287.61 | 1483.33 | 323.27 | 1434.32 | 311.04 |
| RT: How wide? | 1530.88 | 225.05 | 1539.92 | 214.69 | 1523.39 | 217.59 | 1507.19 | 223.63 |

Note: CFP = California Families Project; PGS-E = Pittsburgh Girls Study of Emotion; *SD* = standard deviation.

Figure S2. Emotion × attention interaction effect on reaction times during the facial emotion processing task in the California Families Project (CFP) sample (*N* = 156) and the Pittsburgh Girls Study of Emotion (PGS-E) sample (*N* = 119). Participants rated “How sad does this face make you feel?” and “How wide is the nose?” when viewing four different facial expressions of emotion.

|  |  |
| --- | --- |
| 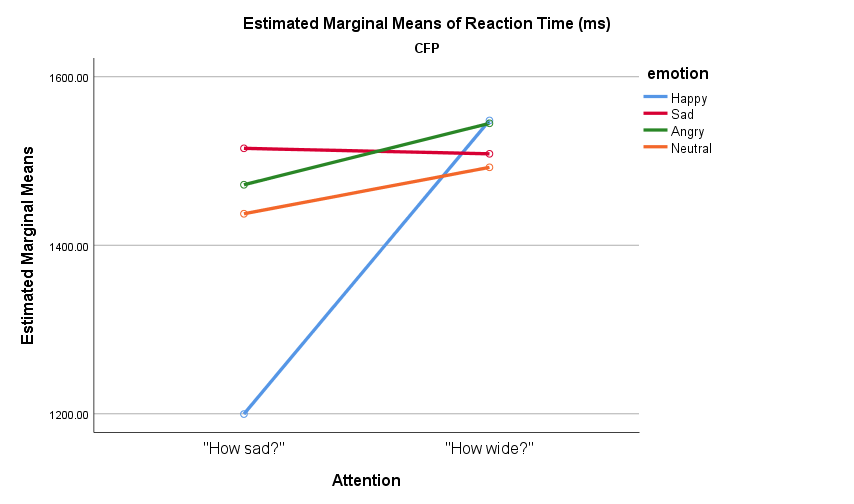 | 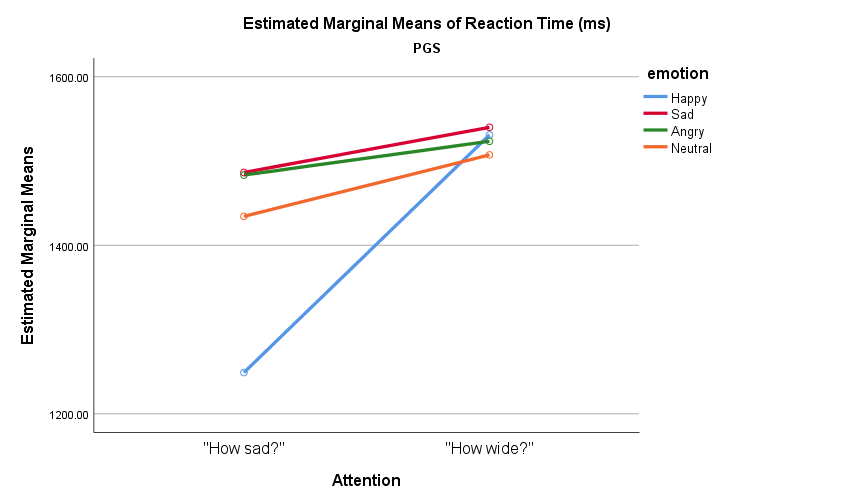 |
|  |  |
|  |  |

Table S3. Mean (SD) percent signal change during sadness introspection for the seven *a priori* defined regions of interest (ROIs) based on a search of ‘self-referential’ in Neurosynth for the California Families Project (CFP) sample (*N* = 156) and the Pittsburgh Girls Study of Emotion (PGS-E) sample (*N* = 119)

|  |  | CFP |  | PGS-E |  |  |  |
| --- | --- | --- | --- | --- | --- | --- | --- |
| **ROI** | **Face Emotion** | *M* | *SD* | *M* | *SD* | *t(273)* | *p* |
| **dmPFC** | happy | -0.05 | 0.21 | -0.05 | 0.21 | 0.27 | 0.786 |
|  | sad | 0.02 | 0.21 | 0.00 | 0.20 | 0.84 | 0.401 |
|  | angry | -0.01 | 0.25 | -0.02 | 0.20 | 0.25 | 0.804 |
|  | neutral | -0.03 | 0.22 | -0.05 | 0.21 | 0.77 | 0.444 |
| **mPFC** | happy | -0.10 | 0.25 | -0.12 | 0.25 | 0.83 | 0.406 |
|  | sad | -0.05 | 0.27 | -0.08 | 0.23 | 0.69 | 0.491 |
|  | angry | -0.11 | 0.29 | -0.11 | 0.23 | 0.08 | 0.934 |
|  | neutral | -0.11 | 0.26 | -0.12 | 0.27 | 0.34 | 0.731 |
| **lTPJ** | happy | 0.00 | 0.20 | -0.10 | 0.27 | 3.58 | < 0.001*^ |
|  | sad | -0.04 | 0.22 | -0.10 | 0.23 | 2.25 | 0.025 |
|  | angry | -0.05 | 0.23 | -0.12 | 0.25 | 2.38 | 0.018 |
|  | neutral | -0.06 | 0.21 | -0.14 | 0.25 | 3.16 | 0.002~ |
| **rTPJ** | happy | 0.00 | 0.20 | -0.09 | 0.20 | 3.56 | < 0.001*^ |
|  | sad | -0.05 | 0.22 | -0.12 | 0.20 | 2.42 | 0.016 |
|  | angry | -0.06 | 0.22 | -0.13 | 0.20 | 2.45 | 0.015 |
|  | neutral | -0.04 | 0.18 | -0.10 | 0.22 | 2.31 | 0.022 |
| **lMTG** | happy | -0.06 | 0.33 | -0.13 | 0.32 | 1.57 | 0.118 |
|  | sad | -0.08 | 0.35 | -0.14 | 0.37 | 1.43 | 0.153 |
|  | angry | -0.09 | 0.34 | -0.15 | 0.32 | 1.63 | 0.105 |
|  | neutral | -0.09 | 0.32 | -0.14 | 0.32 | 1.06 | 0.288 |
| **lITG** | happy | -0.03 | 0.29 | -0.05 | 0.29 | 0.58 | 0.562 |
|  | sad | 0.01 | 0.30 | -0.01 | 0.29 | 0.54 | 0.591 |
|  | angry | 0.00 | 0.29 | -0.02 | 0.30 | 0.64 | 0.522 |
|  | neutral | -0.06 | 0.30 | -0.05 | 0.30 | -0.33 | 0.740 |
| **PCC** | happy | -0.04 | 0.24 | -0.14 | 0.27 | 3.41 | 0.001*^ |
|  | sad | -0.06 | 0.27 | -0.16 | 0.27 | 2.84 | 0.005 |
|  | angry | -0.08 | 0.27 | -0.18 | 0.26 | 3.23 | 0.001* |
|  | neutral | -0.06 | 0.24 | -0.16 | 0.30 | 2.86 | 0.005 |

Notes: dmPFC = dorsomedial prefrontal cortex; mPFC = medial prefrontal cortex; lTPJ = left temporoparietal junction; rTPJ = right temporoparietal junction; lMTG = left middle temporal gyrus; lITG = left inferior temporal gyrus; PCC = posterior cingulate cortex. *Significant at *p* < .0018 (Bonferroni corrected); **^**Remained significant after co-varying gender and expressive suppression; ~Became significant at *p* < .0018 after co-varying gender and expressive suppression.
